# Challenges of using e-health technologies to support clinical care in rural Africa: a longitudinal mixed methods study exploring primary health care nurses’ experiences of using an electronic clinical decision support system (CDSS) in South Africa

**DOI:** 10.1186/s12913-022-09001-2

**Published:** 2023-01-13

**Authors:** Christiane Horwood, Silondile Luthuli, Sphindile Mapumulo, Lyn Haskins, Cecilie Jensen, Deidre Pansegrouw, Neil McKerrow

**Affiliations:** 1 Centre for Rural Health, University of KwaZulu-Natal, Durban, South Africa; 2 Health Systems Strengthening Unit, Health Systems Trust, Durban, South Africa; 3 KwaZulu-Natal Department of Health, Ilembe District, KwaDukuza, South Africa; 4 KwaZulu-Natal Department of Health, Paediatrics and Child Health, Pietermaritzburg, South Africa; 5 Department of Paediatrics and Child Health, University of Cape Town, Cape Town, South Africa; 6 Department of Paediatrics and Child Health, University of KwaZulu-Natal, Durban, South Africa

**Keywords:** Electronic decision-making support system, Integrated management of childhood illness, Mhealth, Child health, IMCI, South Africa, Africa

## Abstract

**Background:**

Electronic decision-making support systems (CDSSs) can support clinicians to make evidence-based, rational clinical decisions about patient management and have been effectively implemented in high-income settings. Integrated Management of Childhood Illness (IMCI) uses clinical algorithms to provide guidelines for management of sick children in primary health care clinics and is widely implemented in low income countries. A CDSS based on IMCI (eIMCI) was developed in South Africa.

**Methods:**

We undertook a mixed methods study to prospectively explore experiences of implementation from the perspective of newly-trained eIMCI practitioners. eIMCI uptake was monitored throughout implementation. In-depth interviews (IDIs) were conducted with selected participants before and after training, after mentoring, and after 6 months implementation. Participants were then invited to participate in focus group discussions (FGDs) to provide further insights into barriers to eIMCI implementation.

**Results:**

We conducted 36 IDIs with 9 participants between October 2020 and May 2021, and three FGDs with 11 participants in October 2021. Most participants spoke positively about eIMCI reporting that it was well received in the clinics, was simple to use, and improved the quality of clinical assessments. However, uptake of eIMCI across participating clinics was poor. Challenges reported included lack of computer skills which made simple tasks, like logging in or entering patient details, time consuming. Technical support was provided, but was time consuming to access so that eIMCI was sometimes unavailable. Other challenges included heavy workloads, and the perception that eIMCI took longer and disrupted participant’s work. Poor alignment between recording requirements of eIMCI and other clinic programmes increased participant’s administrative workload. All these factors were a disincentive to eIMCI uptake, frequently leading participants to revert to paper IMCI which was quicker and where they felt more confident.

**Conclusion:**

Despite the potential of CDSSs to increase adherence to guidelines and improve clinical management and prescribing practices in resource constrained settings where clinical support is scarce, they have not been widely implemented. Careful attention should be paid to the work environment, work flow and skills of health workers prior to implementation, and ongoing health system support is required if health workers are to adopt these approaches (350).

## Background

e-Health is the use of information and communication technologies to support provision of health care, including mobile phones, patient monitoring devices, personal digital assistants (PDAs), desktop computers and other devices [1]. e-Health has been proposed as a way to support healthcare practices and improve quality and access to health care, particularly in low-and-middle-income countries (LMIC) where the need is greatest [2–4]. e-Health includes a variety of approaches targeting patients, health workers or health system support [5]. These interventions include SMS reminders to encourage medication adherence, supportive messages to encourage breastfeeding, support of clinical decision-making, training of health workers, or as a vehicle for record keeping [4, 6, 7]. Health system e-Health interventions can support drug supply management, and scheduling of service delivery [4, 5]. Several of these have been shown to be effective in African settings [4, 5], suggesting that e-Health has potential to provide support for under-resourced health systems. However, there are concerns about scalability, cost-effectiveness, sustainability and lack of information technology infrastructure, skills and support in LMIC settings [5].

Electronic clinical decision-making support systems (CDSSs) are a type of e-Health used to support clinical practice. Clinicians enter clinical data onto the device during the consultation, and the system provides guidance on clinical management. Such systems could support healthcare systems challenged by limited resources, poor health information systems and inadequate numbers of trained staff [3]. Despite successful implementation in high income settings [8, 9], there is a lack of evidence for the effectiveness of CDSSs in LMICs [3, 5, 10], but literature suggests that health workers in LMICs view these approaches positively and are supportive of their use [11]. However, concerns about health system challenges remain, including unrealistic expectations of health workers, conflicts between the knowledge of health workers and the advice provided by the electronic expert system, and increase in workload due to the use of dual systems, as well as costly technical support required to maintain the electronic system [3].

The World Health Organization (WHO), working in collaboration with the United Nations Children’s Fund (UNICEF), developed the Integrated Management of Childhood Illness (IMCI) strategy to guide nurses in the management of common childhood illnesses [12]. IMCI was developed in the 1990s in response to the high numbers of child deaths, and has been adopted in over 75 countries globally [13]. IMCI uses algorithms to provide simple, evidence-based guidelines for managing sick children [14]. Although IMCI evaluations have shown improved outcomes for sick children in some settings [15, 16], results have been mixed with several studies showing poor adherence to IMCI guidelines, with incomplete assessments leading to missed opportunities to provide optimal care [13, 17, 18]. The algorithmic format of IMCI guidelines can be directly converted to a CDSS, with the aim of improving IMCI implementation by ensuring that the algorithm is followed correctly, improving rational prescribing practices and reducing clinical errors.

Several electronic versions of IMCI have been developed with mixed degrees of success [2]. The first of these was an electronic version of IMCI piloted in Tanzania in 2009, using a PDA based system. Findings suggested that electronic IMCI improved adherence to the IMCI protocol and accuracy of IMCI classification [19], and improved the quality of counselling for the mother [20]. More recently, a stepped-wedge trial of electronic IMCI using a tablet-based system in Burkina Faso also showed improved adherence to IMCI guidelines but no improvements in prescribing practices compared to paper IMCI [21]. Qualitative studies suggest that eIMCI was well received, was easy to use and perceived by health workers as a powerful tool to improve care of sick children [22–24].

In South Africa, IMCI has been adapted to include conditions prevalent in the country, with algorithms for identification and management of TB and HIV added to the paper IMCI guidelines [25]. Similar to other settings, an IMCI evaluation in South Africa showed fragmented IMCI implementation [18]. South African IMCI guidelines have been converted into a CDSS for use on desktop computers (eIMCI) and piloted in one district. Findings from the pilot study suggested that eIMCI was acceptable among nurses and caregivers, but showed a low uptake of eIMCI in facilities [23].

In this paper we report the findings of a mixed methods study conducted among newly trained eIMCI nurses to track eIMCI uptake and prospectively explore their experiences of eIMCI implementation in primary health care (PHC) clinics in one district in KZN.

## Methods

We undertook a longitudinal mixed methods study, which was nested within a randomized controlled trial (RCT). The RCT aimed to determine the effectiveness of eIMCI in correctly assessing and managing sick children under 5 years attending PHC services, by comparing the findings of eIMCI and paper-based IMCI practitioners to a gold standard IMCI assessment. The aim of the current study was to track the uptake of eIMCI in clinics, and explore the experiences of eIMCI implementation from the perspective of newly trained eIMCI practitioners. Data were collected using 1) a short quantitative questionnaire to explore participants experience using computers, 2) electronic tracking of eIMCI uptake in participating clinics, 3) a series of IDI’s conducted with selected participants over the implementation period, 4) Focus group discussions (FGDs) with participants after 1 year of eIMCI implementation.

### Study site

The study was undertaken in a predominately rural health district in KZN, South Africa, which was selected in collaboration with KZN Department of Health, because of the strong commitment to IMCI training and implementation in the district. The district covers an area of approximately 3300 sq. km with a population of 657,000 and a population density of 200 people per square Km [26]. 40 percent of the population are under 18 years of age. The district is characterised by high rates of poverty (annual average household income R14 600.00; approx. US$840), and low rates of employment (30.9%). Despite improvement of services in the area, most households in the district do not have access running water inside their dwellings (18.0%) or access to flush or chemical toilets (48.3%) [27].

Health care for sick children is provided by nurses using IMCI protocols at PHC clinics and, when necessary, sick children are referred to district hospitals. At the time of the study, there were 31 PHC clinics in the district, three community health centres, three district hospitals and one regional hospital. Immunization coverage for children under 1 year was 98.3% and vitamin A coverage was 84% [28]. Leading causes of death among children aged under 5 years in the district are neonatal conditions, diarrhoeal disease, lower respiratory infections, malnutrition and HIV/AIDS [26].

### Description of the eIMCI intervention

eIMCI was developed by technical experts as a component of the Virtual Electronic Medical Records (VEMR) system used by the KZN DoH, and was deployed on desktop computers. eIMCI was developed to closely replicate the paper-based IMCI guidelines currently used to manage sick children in clinics (pIMCI). eIMCI practitioners were guided through the consultation and entered the information about the child’s condition on the computer when prompted to do so. All questions and actions were set up to be mandatory, so participants could not proceed with the assessment until all information was provided at each step. On completion of the assessment, eIMCI generated classifications and treatments for each child based on the information entered by the eIMCI practitioner and according to IMCI guidelines. Findings could be printed to provide a clinical record. The number of child consultations where eIMCI was employed was tracked using a function of the eIMCI application.

From 31 PHC clinics in the district, 15 clinics were randomly selected as eIMCI implementation clinics, and computers, printers and eIMCI software were deployed in IMCI consulting rooms in these clinics. One IMCI trained nurse was selected from each clinic to receive eIMCI training, which comprised a one-day training on basic computer skills that were directly required for using eIMCI, including logging in, entering patient information, mouse skills and printing. This was followed by a three-day IMCI update which included a basic review of the IMCI algorithm, followed by a series of case studies and roleplays completed under supervision using eIMCI. After returning to the clinic participants received a minimum of two mentoring visits by an IMCI trainer to support eIMCI implementation, and all participants were certified as IMCI competent.

Extensive technical support was provided to eIMCI trained nurses throughout the study period, with telephonic support available at all times, followed by support visits when required.

### Sampling and recruitment

eIMCI uptake was tracked in all 15 eIMCI clinics throughout the implementation period. All 15 newly trained eIMCI practitioners took part in the study, with nine nurses purposively selected to participate in a longitudinal series of IDIs, based on their computer skills as determined by the telephone survey. Three nurses who scored highest (designated as good computer skills), three who scored lowest (poor computer skills) and three who had a moderate score (moderate computer skills) were contacted by telephone and requested to participate in the IDIs.

### Data collection

All participants completed a telephonic computer skills and self-efficacy questionnaire before eIMCI training. Responses were recorded on a paper data collection tool.

Data collection among 9 purposively selected nurses comprised of a series of in-depth interviews (IDIs) conducted prospectively over the study period. Timepoints were: before eIMCI training (pre-training); after eIMCI training (post-training); 2–3 months after training after completion of mentoring visits (post-mentoring); and after 5–7 months of eIMCI implementation (final). IDIs were conducted in the clinics at a convenient time for participants.

On completion of IDIs, all 15 eIMCI trained nurses were invited to participate in FGDs to further explore enablers and barriers to using eIMCI in the light of the results from eIMCI uptake tracking. FGDs were arranged and participants grouped together according to location to minimize travel. Venues used for FGDs were a district hospital, a community health centre and the Department of Health district offices. No FGDs were conducted in health facilities where participants worked.

All data collection was conducted by two female qualitative researchers (SL, SM), who had extensive experience in conducting qualitative interviews and FGDs, and a masters level research training. Researchers did not have any relationship with participants prior to data collection. All interviews were audio-recorded. IDIs and FGDs were conducted in IsiZulu or English depending on participant’s preference and were conducted privately with only researchers and participants present.

### Data analysis

Data from the telephonic computer skills survey was totalled and is presented as simple frequencies.

IMCI uptake was calculated from the number of consultations using eIMCI as determined from the tracking of the eIMCI application. This is presented as a proportion of all under-5 child consultations in the district each week, obtained from the District Health Information System (DHIS).

All audio recordings of IDIs and FGDs were transcribed verbatim, translated where necessary, and quality checked prior to analysis. Deductive thematic analysis with the aid of NVIVO v12 was used to analyse the data [29]. The researchers who collected the data also analysed the data (SL, SM). Researchers developed a coding framework based on a priori themes from the interview guides, and each individually read a selection of transcripts to confirm the thematic framework. The two researchers then met to discuss and finalise the framework to guide rest of the analysis. The researchers worked closely together to undertake the analysis. The COM-B Theory of Change model was used as the theoretical framework guiding the study, with the assumption that behaviour change is influenced by the interaction of three conditions: capabilities, opportunities and motivation [30].

## Results

At the start of the study all 15 newly trained eIMCI practitioners took part in a telephonic interview about their computer experience and self-efficacy. Demographic characteristics of participants are shown in Table 1. Experience and self-efficacy regarding computer use among participants is shown in Table 2.

**Table 1:** Sociodemographic characteristics of participants

	*N* = 15
Age	Median = 42
	Age range = (26–58)
Race	
African	14
Indian	01
Gender	
Male	02
Female	13
How long ago were you trained in IMCI?	
1 to < 3 years ago	05
3 to < 6 years ago	03
More than 6 years ago	06
Cannot remember	01

**Table 2:** Self-reported computer experiences and self-efficacy among participants

Computer competency questions ***N*** = 15		
When was the last time you used a computer?		
Within last month	06	
1–12 months	03	
> 12 months	04	
Never	02	
How confident do you feel about using a computer?		
Not very confident	06	
Somewhat confident	05	
Very confident	03	
Missing data	01	
Owns their own computer	03	
	Yes	No
Have you used a computer this week?	02	13
Do you have an email address?	12	03
Do you know how to send an email from a computer (not your phone)?	07	08
Have you ever used a computer to write assignment or similar?	11	04
Are you able to search and find website on the internet using computer?	09	06
Can you use Microsoft word to write a letter on the computer?	07	08
Are you able to create folders on the computer?	05	10
Are you able to save documents into different folders?	06	09
Are you able to find saved documents on the computer?	07	08
Are you able to print documents from the computer?	09	06

A total of 36 IDIs were conducted between October 2020 and May 2021 among nine nurses (four IDIs per participant). IDIs were between 10 and 30 minutes in duration.

Three FGDs were undertaken in October 2021 with 11 participants, of whom eight had participated in the IDIs. Three participants were unavailable to participate and one participant refused. Duration of FGDs was between 107 and 117 minutes. Participant numbers were allocated to individuals and these were maintained across the IDIs and the FGDS.

### Uptake of eIMCI in facilities

eIMCI uptake was tracked during the implementation period in all participating clinics using a function of the eIMCI application, and is shown in table 3 as a proportion of all under 5 children attending all 15 participating clinics in the district. It was optional for eIMCI practitioners to use eIMCI for children attending for well child visits, and eIMCI practitioners were not always on duty, so while this table provides an indication eIMCI uptake in participating clinics it is not intended to be an exact representation of uptake by individual eIMCI practitioners.

**Table 3:** The proportion of all consultations with children aged < 5 years where eIMCI was used in each participating clinic

Year	2020	2020	2020	2021	2021	2021	2021	2021	2021
Month	Oct	Nov	Dec	Jan	Feb	Mar	Apr	May	Jun
**Clinic1**	7,5%	16,6%	36,7%	0,0%	30,2%	63,8%	66,5%	58,6%	7,5%
**Clinic 2**	3,2%	18,6%	22,5%	14,7%	11,7%	10,4%	9,8%	2,9%	3,2%
**Clinic 3**	13,3%	17,2%	12,8%	4,5%	4,1%	21,5%	1,4%	5,6%	13,3%
**Clinic 4**	29,7%	23,7%	35,7%	23,6%	21,0%	30,1%	15,2%	23,4%	29,7%
**Clinic 5**	1,7%	3,2%	2,5%	0,0%	0,9%	0,3%	0,5%	2,0%	1,7%
**Clinic 6**	12,5%	44,6%	48,3%	56,7%	61,7%	57,0%	58,3%	61,2%	12,5%
**Clinic 7**	0,0%	24,4%	14,0%	4,5%	0,0%	9,7%	16,1%	7,4%	0,0%
**Clinic 8**	11,7%	14,2%	11,3%	9,3%	6,1%	4,4%	5,7%	3,0%	11,7%
**Clinic 9**	1,1%	10,0%	14,1%	5,4%	23,6%	24,2%	34,0%	32,8%	1,1%
**Clinic 10**	2,4%	4,8%	0,0%	8,1%	24,3%	26,1%	29,6%	10,4%	2,4%
**Clinic 11**	11,8%	20,3%	5,5%	4,1%	0,2%	30,2%	4,8%	1,0%	11,8%
**Clinic 12**	5,4%	6,3%	19,8%	18,1%	6,7%	26,4%	51,9%	42,3%	5,4%
**Clinic 13**	3,2%	6,7%	3,8%	1,7%	0,0%	1,1%	0,3%	0,0%	3,2%
**Clinic 14**	0,1%	1,3%	1,1%	3,1%	3,4%	7,3%	0,2%	0,0%	0,1%
**Clinic 15**	0,0%	2,4%	1,3%	23,4%	11,0%	25,2%	23,1%	5,8%	0,0%

### Experiences of eIMCI implementation

Overall, nurses reported positive experiences of using eIMCI. Most participants stated that they found eIMCI to be very helpful, simple, quick, and accurate in the management of sick children. Participants mentioned that eIMCI improved the quality of assessments and that using eIMCI gave them confidence ‘*eIMCI gives that confidence as a nurse that you managed this sick child and did everything’ (P3, FGD 3).* Participants mentioned that eIMCI guided them step-by-step through the examination of the child, including growth monitoring, prevention of mother-to-child transmission of HIV (PMTCT) and the management of sick children requiring hospital referral. In addition, participants highlighted that the mandatory questions ensured that assessments were comprehensive.

*It [eIMCI] is fast and another good thing is it takes you from the beginning to the end where it will come up with the treatment. It [eIMCI] tells you if you have omitted something. You do not go into the next assessment without filling in other things. You need to fill everything, it [eIMCI] tells you that you don’t have to continue, when there is an error it writes in red. So, I think an advantage is that it [eIMCI] makes you do a thorough, thorough assessment, thorough treatment, and thorough referral (P4, post-training IDI).*

Most participants mentioned that eIMCI was well received in their facilities. Their colleagues were interested to learn about eIMCI, and as a result sometimes asked for assistance from the eIMCI practitioner if they had a complicated case.

*They [colleagues] were happy and each and every one was eager to see how will this go, so they were positive just to see the change. If you show them a printout and they see that it tells you how to act, the child is this age, this is the dose and all that. You just show them everything that is in the printout so they can see that this thing makes everything easy, because sometimes with the old method or with the old system you end up omitting things. (P4, FGD1).*

### Challenges to eIMCI implementation

Although the nurses appreciated and praised eIMCI, they reported several challenges which affected how eIMCI was implemented, and reduced their ability to transfer their skills to the workplace and establish themselves as confident eIMCI practitioners. Many participants reported not using eIMCI consistently during consultations with sick children, and reverting to pIMCI when challenges arose.

Reported challenges to eIMCI implementation are presented in themes as follows: lack of computer skills among eIMCI practitioners; difficulties integrating eIMCI processes into routine consultations; lack of health system support for eIMCI implementation; and poor alignment of eIMCI with other priority clinic programs.

### Lack of computer skills among eIMCI trained nurses

Most nurses reported having minimal experience and skills with computers prior to starting eIMCI (Table 2), and the one-day computer literacy component of the eIMCI training aimed to equip participants only with the basic computer skills required to use eIMCI. Poor computer skills had a wide-ranging impact on eIMCI implementation, affecting participants’ ability and confidence as they started to use eIMCI in the health facility. In particular, participants reported that eIMCI consultations took longer, which was a major barrier to adopting eIMCI in busy clinics.

*To capture a child in a computer it’s not as easy as you may think because it takes more time, in such a way that it’s can take 30 to 40 minutes if you are going to do it properly. Whereas if I ask the mother what is wrong with your child and they say they are feeling hot, coughing. Then I write that down, the mother said the child is coughing and has fever, then the diagnosis is flu, then prescribe the prescription amoxicillin and Panado. It [eIMCI] is not as easy as that (P3, FGD 3).*

Nurses reported that there were frequent technical challenges, many of which were straightforward and should have been easy to resolve, but were exacerbated by poor computer skills. Several participants expressed that eIMCI training failed to equip them with all the skills required to operate a computer, and they did not have skills to address the technical challenges that arose. For example, a common challenge highlighted by participants was with logging into eIMCI, particularly logging in again after a break. Each time eIMCI was left for a short period a new login was required, which added to the perception that eIMCI was time consuming. As a result, participants would return to using pIMCI which was quicker for them and where they were more confident and could take shortcuts. This participant highlights the time it takes to gain confidence to address technical concerns.

*Like for example today I could not login but yesterday I was able to login, it says I am already logged in and I spent like 30 minutes trying and I gave up. So, there are those technical issues, if only it [eIMCI] is a normal program where you just click to login and continue with the work. Avoid stumbling blocks like [having] to go and clear logs (P10, FGD1).*

Other technical challenges included not being able to find a child who had previously been entered on the eIMCI system, and restarting the computer after a power outage.

*Then after searching [for] a child, after it has come up, if I am trying to get into the previous information it can’t open … it doesn’t allow me to access the previous information. The child’s name comes up as a child who was previously registered but you cannot access the information. Then I end up entering [the child] as a new [child]. (P4, mentoring visit).*

Extensive IT support was provided during eIMCI implementation but poor computer skills affected the functioning of the IT support. Participants were frequently unable to follow telephone instructions, so that even minor technical challenges required an IT support visit, with the result that eIMCI was unavailable for periods of time. Calling for support was time consuming for eIMCI nurses, and reduced nurse’s confidence and willingness to use eIMCI. In a few cases, issues would go on for days without being resolved while nurses returned to using paper IMCI.

*Some of us are not computer literate so even if it’s a telephonic (advice) you may not understand. So, for me it’s better if they have a face-to- face support (visit) (P6, Final IDI).*

### Poor integration of eIMCI into consultation

eIMCI was designed to guide the consultation step-by-step. Practitioners were prompted to undertake the clinical assessment, and enter the clinical findings as they went along. However, most nurses did not use eIMCI as intended, instead using different strategies to incorporate the use of eIMCI into their consultations. Most participants reported starting by having a conversation with the mother about the sick child, asking questions based on IMCI, and doing the examination, and only logged into eIMCI once this was completed.

*As time went on I was able to ask questions that are now going to pop up there [on eIMCI] before I even start to log in. So, I’ll be asking questions like, observing, assessing before I even go to eIMCI. Then I know that this is going to pop up, I’m going to need this later… it hasn’t changed for me, like I always ask questions. I’m always gathering as much information as possible. (P5, Final IDI).*

Participants reported that when the mother did not know the answer to the questions or the carer was not the mother, they were unable to move forward with the assessment. eIMCI is set up with mandatory questions throughout, without the option to enter that the information is unknown. This was a challenge and caused delays, undermined participants confidence in eIMCI, and in some cases, nurses admitted that they would guess or make up the answers.

S*ometimes the granny comes with the child and tells you that this child came with her father from Johannesburg, she doesn’t have a card and she doesn’t know when the child was born and [you] end up assuming that this child is this age and she really don’t know when this child was born ... But when they are in front of you, you are just guessing because she doesn’t know anything, she doesn’t know about child’s immunizations (P2, FGD1).*

Another concern expressed by nurses was the quality and completeness of the eIMCI printouts. Printouts were intended to provide the documentation to be kept in the child’s clinical records. Nurses reported that the printouts often appeared incomplete, with some classifications missing, this undermined the confidence that nurses had in the eIMCI system overall. Nurses would spend extra time during the consultation filling in the missing information on the printouts, which created work and caused further delays.

*We have a problem, in the first page it asks you ‘is it is a sick child or well child?’. Then if it is a sick child then we have to write the problem but on the printout there is nothing [shown]. When referring the sick child and on your printout you have to write all the problems and you have also typed in the treatment at the end, the treatment will come out but the problem is not on the printout. (P5, FGD2).*

### Lack of health system support

There was shortage of staff reported in most clinics, and, as a result, eIMCI nurses were frequently unable to consult only with sick children but also had to see other patients in the queue, for example pregnant women or chronic patients. This made it difficult to use eIMCI consistently, particularly because each time a child came into the consulting room a new login was required, making it easier to use pIMCI.

*Where I work from, I don’t work with children only, I also attend pregnant women. You will find that after I finish attending a child the next patient would be a pregnant woman coming for antenatal care, then I will attend her. Then the screen will lock. When I go back to login the system will say I have already logged in, then I will end up going to clear logs (P3, FGD1).*

Staff shortages also led to frequent staff rotations, with nurses being allocated to work where there was no computer available or where they were not consulting with sick children, despite being newly trained in eIMCI. As a result, nurses would go for long periods without using eIMCI and were unable to effectively transfer their new skills to the workplace.

*I do not do it [use eIMCI] every day, because we are busy. I am at a very busy clinic so when I do get a chance I do it [use eIMCI] ….. When I am not in IMCI I am here at chronic [seeing chronic patients] because there is shortage this side. I go there [to the chronic section] to close a gap. (P4, post-mentoring IDI).*

Another challenge described by participants was a lack of support from their colleagues because eIMCI slowed down their work. One participant stated that her colleague was unsupportive and would slow down her own consultations because the colleague perceived that she was doing more of the work. The unsupportive work environment was a source of added pressure, prevented nurses from gaining eIMCI skills, and reduced their confidence using eIMCI.

*Then initially, at the beginning of the [eIMCI] program my manager was alright but I realized that my colleague was not okay because initially it took [me] a while to complete a patient [using eIMCI] because I was still learning. She would also take her time on the other room, do things slowly so that I would end up taking sick patients. (P2, FGD1).*

Whenever there was a heavy workload in the clinic many nurses opted to go back to pIMCI because they felt that they could not work fast enough using eIMCI. For example, one eIMCI trained nurse would see a few patients using eIMCI then switch back to pIMCI because it was faster and she was more comfortable using paper. Since she knew the algorithm by heart she could then skip some components of the IMCI guidelines to save time, which was not possible with eIMCI. This nurse had poor computer skills and was frequently overwhelmed by work and found it challenging to use eIMCI.

*Sometimes I start to enter the baby into the computer, when there is an emergency of a sick baby, I end up stopping there and consult with that sick one. When I come back I look at the time, the mother is waiting for me and that’s where I am not continuing with the sequence [eIMCI]. I just look at the problem and I address the problem, then I give the mother the Road to Health card, I write all the things they need and say “go”. There is no time to look at the formality of ticking and ticking or using the computer to do the things, I just tick the important things that I know. (P9, FGD 2).*

### Poor integration of eIMCI with other priority clinic programs

In the clinics a variety of clinical programmes were operating with particular requirements for monitoring and record keeping. During eIMCI implementation the Ideal Clinic Programme was being assessed, and was therefore being closely supervised and audited. However, eIMCI did not comply with the record-keeping requirements, so nurses had to do additional administrative work in order to reach the standard required for their Ideal Clinic assessments. Participants expressed concerns that the additional administrative work added to the consultation time and contributed to eIMCI nurses choosing to use pIMCI.

*If you look at eIMCI it doesn’t include everything that is required by Ideal Clinic, meaning you have to do a triple job. You have to go straight into the computer to fill the information of the patient’s [history], then you write on the Road to Health Card [patient held record], thereafter you have to write on the clinical chart of the patient. You do all those things but when you fill the information in the computer now, not that you fill everything, the computer is just specific, it requires [only] what is needed on it. That is a big challenge but if you take the checklist for Ideal Clinic, like if you are doing file audit…. you took some 10 files just to see if we can score 100% but we never scored 100% because some of the questions are not there [in eIMCI]. (P3, FGD Group 3).*

## Discussion

eIMCI is a CDSS based on IMCI, a well-established approach to management of sick children that has been implemented for decades in South African clinics. The purpose of a CDSS is to assist clinicians making complex decisions, such as those required when managing sick children, to improve adherence to evidence-based guidelines, ensure complete and standardised assessments, and improve prescribing practices [2]. We report uptake of eIMCI in the early implementation period, together with contemporaneous experiences of newly trained eIMCI practitioners, and our findings demonstrate that, despite positive attitudes expressed by nurses, eIMCI uptake was low. Tracking data obtained from the eIMCI application showed wide variations in eIMCI uptake between clinics and over time, with several clinics rarely using eIMCI. Several electronic versions of IMCI have been evaluated in LMICs, the first of these more than a decade ago [19, 20], and since then several versions of eIMCI have been shown to effectively improve adherence to IMCI guidelines and improve prescribing practices [2, 21, 31, 32]. However, despite their potential for improving outcomes for children, no version of eIMCI has been implemented at large scale [2]. Our findings shed light on possible reasons for this, highlighting the need for wide ranging changes at individual, facility and health system levels to support the implementation of a new CDSS, if this is to be successfully adopted at scale [4].

A number of challenges were highlighted by newly trained eIMCI practitioners as barriers that prevented them from using eIMCI and gaining skills and confidence as eIMCI practitioners. Our findings suggest that newly trained eIMCI practitioners with little or no computer skills struggled to transfer their new skills to the workplace because eIMCI took longer and presented them with technical challenges that were difficult and time-consuming to address. In addition, eIMCI disrupted their work flow and created a higher administrative burden. Instead of receiving support from the health system to allow them to focus on developing competency, eIMCI practitioners were faced with heavy workloads, long queues, lack of support from colleagues and frequent deployment away from the IMCI clinical area. eIMCI printouts that were intended to provide a clinical record were frequently incomplete and not aligned with requirements for record keeping for other programmes, further undermining confidence of nurses and supervisors in the eIMCI expert system. We contend that this approach to eIMCI implementation, which focused largely on capacitating individual health workers, was almost certain to fail.

Introduction of a CDSS or other e-Health initiative should be managed as a health system strengthening intervention, and be integrated into all existing systems and structures. This requires individuals, facilities and the health system to change, in particular health workers have to change the way they think and work [33]. Adopting a CDSS requires inevitable workflow adjustments for health workers, which should be actively managed and addressed, for example our participants noted that making questions compulsory affected their ability to navigate the system. Achieving changes to health worker practices is challenging for any new program, and integrating e-Health into daily care processes is complex and requires specific inputs to achieve the required change [30]. Further, implementing a new programme requires co-ordination and collaboration between different levels of the system to ensure that the intervention is supported and aligned with other clinic programs [33]. In our study poor alignment of eIMCI with other clinic programs had implications for nurses’ workload and on the support for eIMCI from different structures within the health system, and acted as a disincentive to using eIMCI.

Many CDSSs fail, even in high income settings, and e-Health interventions are failing to achieve the foreseen benefits [34]. More research is required to explore how digital and traditional health care can blend in the long term [33]. The CDSSs that are most likely to be adopted are those which provide decision support at the time it is required, with minimal disruption of clinicians work flow, and provide clear recommendations rather than a range of advice [35]. Further, effectiveness of the CDSS was enhanced when clinicians were required to document a reason for deviating from recommendations [35]. The development of eIMCI aimed to make the system user-friendly, aligned with the familiar actions of pIMCI, and provided clear guidance on management, counselling and referral of sick children. In addition, eIMCI removed the need for cumbersome paper booklets and the requirement to classify and treat the child based on multiple algorithms. However, the use of compulsory questions, intended to improve adherence to guidelines, reduced the flexibility of the consultation, disrupted the workflow for practitioners and failed to make allowance that some clinical information may be unavailable. In addition, our findings suggest that nurses did not use eIMCI as intended, rather completing the clinical assessment from memory before logging on to eIMCI, likely leading to errors and shortcuts. e-Health interventions that are well aligned to existing patterns of work are most likely to be adopted, and changes in workflow are the most common reason for failure of e-Health interventions [33, 34]. However, the requirement to change practices can also be seen as an opportunity to improve efficiency and redistribute tasks [34]. We suggest that additional formative research on existing IMCI work practices and how best to align eIMCI with these practices, as well as training and mentoring specifically focused on how to integrate eIMCI into the consultation would improve implementation.

Lack of computer skills was a strong theme highlighted by all participants. In PHC settings in South Africa computers have an administrative function and have not been used to support clinical practice, thus using computers during consultations was unfamiliar to clinic nurses. Poor computer literacy had wide-ranging impacts on nurse’s ability to effectively implement eIMCI and was an underlying factor for many challenges reported. Technical and usability issues are key to uptake of CDSSs and inflexibility and complexity of technology are a barrier to uptake in all settings [33]. eIMCI nurses received very basic computer training aimed at giving them the skills to use eIMCI but no additional computer skills, with the result that all technical challenges became major barriers to uptake. Poor computer skills and infrastructure are common barriers to implementation of electronic interventions in African settings [11], but can also be an opportunity to leapfrog older technologies and implement newer, cheaper and more user-friendly technology. The use of desktop computers to deploy eIMCI, as suggested by the DoH, was cumbersome and not user-friendly. The use of mobile devices with touch screens would have allowed nurses to use eIMCI in all areas of the clinic and would have been more familiar to nurses most of whom use smartphones on a daily basis.

### Strengths and weaknesses

This study employed a strong prospective mixed methods design, which allowed for in-depth understanding of the experiences of nurses using eIMCI in real time during the early months of implementation. Monitoring uptake of eIMCI over the same time period allowed researchers to provide feedback and challenge participants during the FGDs. While providing in-depth insights of nurses’ experiences, the qualitative design does not allow findings to be generalized. In addition, the proportion of children assessed using eIMCI was generated using a function of the eIMCI application, and gives an indication of eIMCI uptake but does not allow for accurate measurement of health worker performance or statistical analysis of uptake by individual practitioners.

## Conclusion

This study reports the challenges experienced by newly trained eIMCI practitioners as they returned to the workplace to implement a computer-based decision support tool based on IMCI. Our findings highlight that a CDSS should be seen as a whole health system change, requiring multi-level support and broad ranging changes to the way people work. Further, it is important to focus on making the system user-friendly, using technology that health workers are familiar with and allowing that information may be unavailable. We suggest that despite the challenges, eIMCI has the potential to improve quality of care for sick children in low resource contexts, if careful consideration is given to alignment of eIMCI to clinical practices at individual, facility and health system levels. Further research is required to understand and overcome constraints to adopting eIMCI and other CDSSs at scale in LMICs.

## Data Availability

The datasets used are not currently available because further analysis is underway but are available from the corresponding author upon reasonable request.

## Abbreviations

eIMCI: Electronic Integrated Management of Childhood Illness
CDSS: Electronic clinical decision support system
FGD: Focus group discussion
IDI: In-depth interview
IMCI: Integrated Management of Childhood Illness
LMIC: Low- and middle-income countries
pIMCI: Paper-based IMCI
PDA: Personal digital assistant
PMTCT: Prevention of mother-to-child transmission of HIV
PHC: Primary health care
UNICEF: United Nations Children’s Fund
WHO: World Health Organization
